# Expiratory Flow Bias and Physiological Effects of Rapid Chest Compression in Mechanically Ventilated Neurocritical Patients: A Secondary Analysis of a Randomized Controlled Trial

**DOI:** 10.3390/jcm14186516

**Published:** 2025-09-16

**Authors:** Ricardo Miguel Rodrigues-Gomes, Rosa Martinez Rolán, Maribel Botana-Rial, Alejandra Del Río González, Eduardo Arán-Echabe

**Affiliations:** 1 Facultade de Medicina, Universidad de Santiago de Compostela, 15702 Santiago de Compostela, Spain; 2 Galicia Sur Health Research Institute (IIS Galicia Sur), SERGAS-UVIGO, 36312 Vigo, Spain; 3 Neurosurgical Service, Álvaro Cunqueiro Hospital, 36312 Vigo, Spain; rosamartinezrolan@gmail.com; 4 Pulmonary Department, Alvaro Cunqueiro Hospital, EOXI, 36312 Vigo, Spain; maria.isabel.botana.rial@sergas.es; 5 PneumoVigoI+i Research Group, Sanitary Research Institute Galicia Sur (IISGS), CIBERES-ISCIII, 36312 Vigo, Spain; 6 Physiotherapy Department, Hospital Álvaro Cunqueiro—SERGAS, 36312 Vigo, Spain; alejandra.del.rio.gonzalez@sergas.es; 7 Surgery Department, Universidad de Santiago de Compostela, 15705 Santiago de Compostela, Spain; eduardo.aran.echabe@usc.es; 8 Neurosurgery Service, Universitary Clinic Hospital, 15706 Santiago de Compostela, Spain

**Keywords:** expiratory flow bias, respiratory physiotherapy, airway clearance techniques, mechanical ventilation, intracranial pressure

## Abstract

**Background:** Mechanical ventilation compromises airway clearance, with expiratory flow bias (EFB) being a critical determinant of mucus transport. The rapid chest compression technique (RCCT) generates high EFB, yet evidence in neurocritical patients is limited due to concerns regarding intracranial pressure (ICP). This secondary analysis of a randomized controlled trial examined the effects of RCCT on ventilatory mechanics and physiology in acute brain-injured patients under invasive ventilation. **Methods:** Fifty neurocritical patients were randomized to RCCT (Intervention) or passive leg mobilization (Control). RCCT was applied bilaterally during expiration once every three respiratory cycles for 5 min; controls underwent 5 min of passive cycling. EFB, derived from inspiratory and expiratory peak flows, was assessed at baseline (T–5), during intervention (T0–T5), and post-intervention (T+5, T+30). Arterial blood gases, mean arterial pressure (MAP), heart rate (HR), and ICP were also analyzed. Group comparisons used parametric/non-parametric tests; associations were explored via Spearman’s rho. **Results:** Baseline EFB did not differ between groups. From T0 to T5, Intervention patients showed significantly higher EFB (all *p* < 0.001). PaCO_2_ decreased within the Intervention group (*p* = 0.015) but not in controls (*p* = 0.601). No between-group ΔPaCO_2_ differences emerged. At T5, HR correlated negatively with EFB (ρ = −0.49, *p* = 0.013). No associations were found with age, sex, lesion type, MAP, or ICP. **Conclusions:** RCCT effectively increased EFB in ventilated neurocritical patients without affecting ICP, supporting its safety and potential role in airway clearance.

## 1. Introduction

Mechanical ventilation (MV) is an indispensable, life-sustaining intervention for critically ill patients. However, it frequently leads to a significant impairment of the primary mechanisms of airway clearance, affecting both peripheral processes, such as mucociliary transport and gas–liquid interaction, and central mechanisms, including the cough reflex. This widespread mucociliary dysfunction and suppressed cough reflex are highly prevalent among intubated and sedated patients. The resulting retention of pulmonary secretions is a major complication, contributing to an increase in airway resistance, partial or total airway obstruction, alveolar hypoventilation, atelectasis, hypoxemia, and increased work of breathing. Moreover, it creates a favorable environment for bacterial proliferation and the development of pulmonary infections, including ventilator-associated pneumonia (VAP), which in turn prolongs MV duration, increases the length of stay in the Intensive Care Unit (ICU), and worsens patient prognosis [1,2,3,4].

In this challenging clinical scenario, respiratory physiotherapy (RP) and airway clearance techniques (ACTs) are fundamental for maintaining airway patency and improving patient outcomes. Given the often-altered cough reflex in ventilated patients, the physiological principle of flow bias emerges as a crucial mechanism for the effective mobilization and removal of secretions. Flow bias is defined as the difference between the peak inspiratory flow (PIF) and the peak expiratory flow (PEF) during gas movement within the airways. An expiratory flow bias, where the PEF is greater than the PIF, is essential as it promotes the cephalic movement of mucus towards the glottis. The efficacy of mucus transport via this two-phase gas–liquid flow mechanism is influenced by critical factors such as inspiratory-expiratory air velocity, mucus viscosity, and the thickness of the mucus layer. Various thresholds have been described for effective cephalad mucus displacement: early descriptions suggested a PEF:PIF ratio greater than 1.11, followed by in vitro studies indicating a PEF-PIF difference exceeding 17 L/min as a critical threshold for mucus movement. More clinically relevant in vivo research in mechanically ventilated pigs, using their own mucus, identified a PEF-PIF difference greater than 33.0 L/min (associated with an average PEF:PIF ratio of 4.3) as significant for central mucus displacement [3,4,5,6,7,8].

Optimizing this expiratory flow bias is fundamental for enhancing mucus displacement and the effectiveness of various ACTs, including ventilator hyperinflation, expiratory rib cage compression (ERCC), PEEP-ZEEP maneuvers, and mechanical insufflation-exsufflation (MI-E). Among these, the rapid chest compression technique (RCCT) is specifically designed to increase expiratory flow and facilitate the transport of mucus out of the bronchial tree. Studies have shown that RCCT can effectively generate a significant expiratory flow bias, reaching approximately 50 L/min, which is well above the empirically defined threshold for mucus mobilization. The PEEP-ZEEP maneuver alone can also generate an expiratory flow bias sufficient for secretion removal (e.g., a mean PEF-PIF difference of 49.1 ± 9.4 L/min), and combining it with manual chest compression (MCC) further increases this bias [3,4,9,10,11].

Despite the physiological plausibility and promising results from in vitro and animal models, there remains a dearth of robust human evidence on the safety and effectiveness of RCCT in critically ill, mechanically ventilated patients, particularly those with acute brain injuries (ABI). The application of chest physiotherapy techniques in neurocritical patients has traditionally been approached with caution due to concerns about potential increases in intracranial pressure (ICP). Previous studies investigating chest physiotherapy in ABI patients have yielded mixed results regarding its impact on ICP, with some reporting no significant changes, while others found transient increases in ICP. A critical limitation of many earlier studies is the common inclusion of tracheal suctioning in the intervention protocols, which is independently known to cause significant reflex increases in ICP and mean arterial pressure (MAP), thereby confounding the true effects of the physiotherapy maneuvers themselves. Indeed, manual chest percussion has been shown to raise ICP compared to mechanical vibration [12,13,14,15].

This paper presents a secondary analysis of the randomized controlled trial conducted by Rodrigues-Gomes et al. (2025) [14]. The primary study investigated the effects of the rapid chest compression technique on intracranial and cerebral perfusion pressures in acute neurocritical patients. Importantly, that trial found that the RCCT did not increase ICP during its application or even 30 min after it. This secondary, exploratory analysis builds on those findings by specifically examining the detailed flow bias and ventilatory changes generated by RCCT in this vulnerable population. To our knowledge, this is the first study to evaluate RCCT in mechanically ventilated neurocritical patients with continuous ICP monitoring. By addressing this evidence gap, we aim to highlight both the safety and physiological potential of RCCT for airway clearance and to provide a rationale for larger, multicenter clinical trials [14].

## 2. Methods

This study is a secondary analysis of a randomized, single-blinded, single-center controlled trial in acute neurocritical patients under invasive mechanical ventilation (ClinicalTrials.gov NCT03609866). The original trial, conducted at Hospital Álvaro Cunqueiro (Vigo, Spain), has been previously described in detail, including sample size calculation, inclusion and exclusion criteria, and primary results. The protocol was approved by the regional ethics board (2018/446), approval date is 20 November 2018.

Fifty patients with severe acute brain injury (traumatic brain injury, ischemic stroke, subarachnoid hemorrhage, or intracerebral hemorrhage) were randomized to Intervention (rapid chest compression technique, RCCT) or Control (passive lower-limb mobilization). RCCT was applied bilaterally during the transition from inspiration to expiration, once every three respiratory cycles for 5 min, with the aim of generating an expiratory flow bias ≥ 33 L/min. All RCCT procedures were performed by senior ICU physiotherapists routinely applying this technique in their daily clinical practice with critically ill patients. The control intervention consisted of motorized passive leg cycling for 5 min. Both procedures were performed with patients in supine position and 30° head elevation. To ensure standardized ventilatory conditions for expiratory flow bias assessment, all patients were switched from their baseline ventilatory mode to volume-controlled ventilation with a square flow pattern one hour before the intervention. This adjustment was part of the study protocol and was maintained throughout the intervention period. Safety monitoring included heart rate, blood pressure, intracranial pressure (ICP), cerebral perfusion pressure, and oxygen saturation.

For this secondary analysis, the primary outcomes were ventilatory mechanics and expiratory flow bias, derived from inspiratory and expiratory peak flows at predefined time points: T–5 (baseline), T0, T1–T5, T+5, and T+30. Additional variables included arterial blood gases, mean arterial pressure, heart rate, and ICP. Radiological data such as chest X-ray findings were not systematically collected or analyzed in this study. Descriptive statistics were calculated, normality was tested with Shapiro–Wilk, and between- and within-group comparisons were performed using *t* tests, Mann–Whitney U tests, ANOVA, or Friedman tests as appropriate. Correlations between expiratory flow bias and physiological variables were assessed using Spearman’s rho. A *p*-value < 0.05 was considered significant.

**Figure 1:**
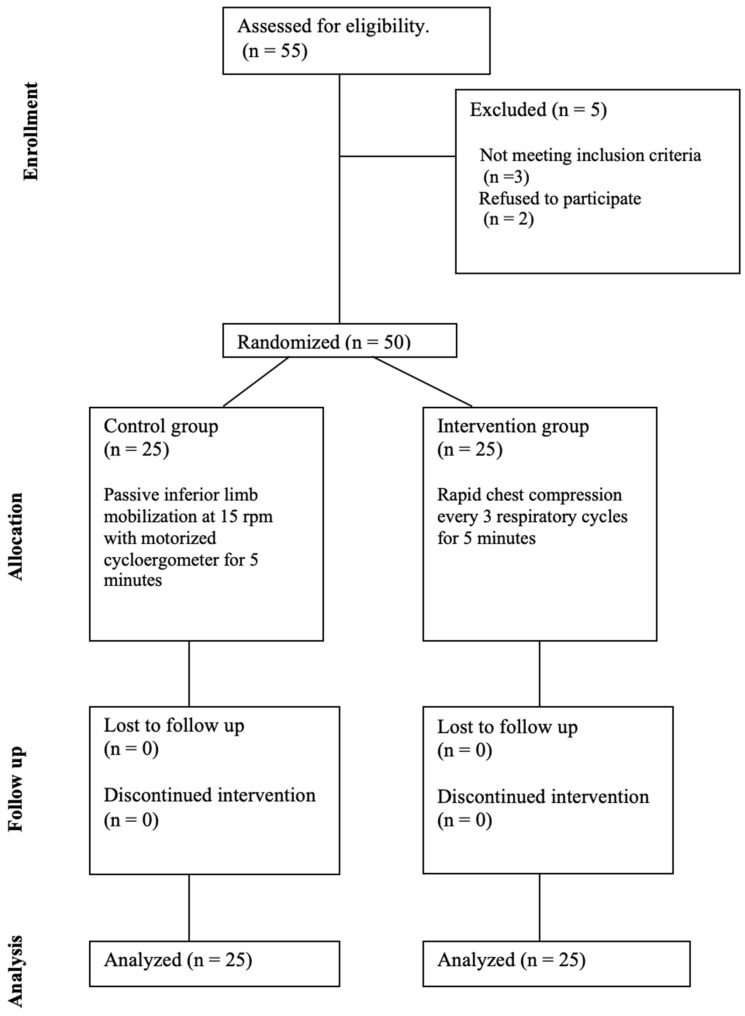
CONSORT flow chart.

The CONSORT flow chart of patient inclusion and randomization can be seen in Figure 1 [14].

## 3. Results

Between May 2021 and December 2023, the recruitment process assessed 55 possible subjects; 5 were excluded—3 did not meet inclusion criteria and 2 declined to participate—resulting in the allocation of 50 participants.

Baseline demographic and clinical characteristics of the study population are summarized in Table 1.

### 3.1. Expiratory Flow Bias Across Groups

**Figure 2:**
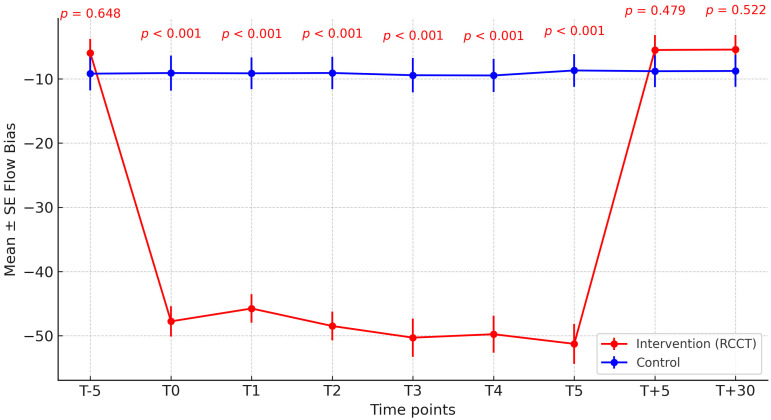
Mean expiratory flow bias at each time point.

At baseline (T–5), expiratory flow bias (EFB) values did not differ significantly between groups (*p* = 0.65). From T0 to T5, the Intervention group demonstrated significantly higher EFB compared with the Control group (all *p* < 0.001). These differences were consistent across boxplots and trend analyses, suggesting that the physiotherapy maneuver effectively increased EFB in the Intervention group (Figure 2).

### 3.2. Association with PaCO_2_

**Figure 3:**
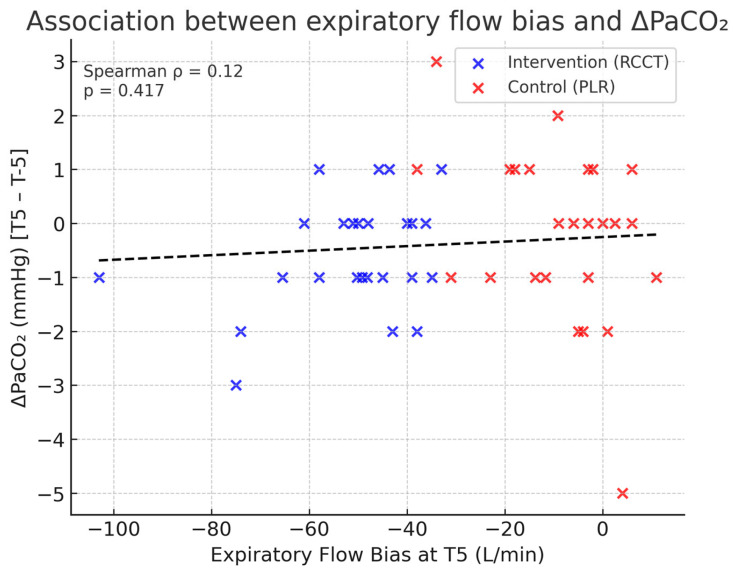
Correlation between expiratory flow bias at T5 and PaCO_2_ changes.

In the Intervention group, PaCO_2_ decreased significantly between paCO2-5 and paCO2+5 (Wilcoxon *p* = 0.015), while no significant change was observed in the Control group (*p* = 0.601). Between-group comparison of ΔPaCO_2_ did not reach significance (*p* = 0.241). Correlation analyses revealed no significant association between ΔPaCO_2_ and EFB at T5 (ρ = 0.12, *p* = 0.417) (Figure 3).

### 3.3. Influence of Sex and Age

**Figure 4:**
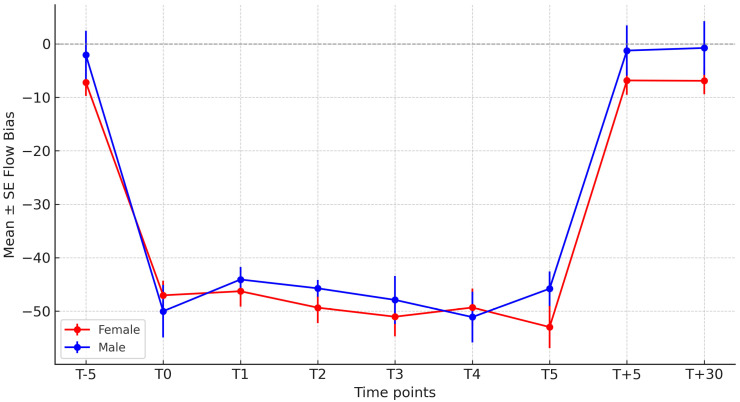
Expiratory flow bias at each time point in the intervention group, stratified by sex.

**Figure 5:**
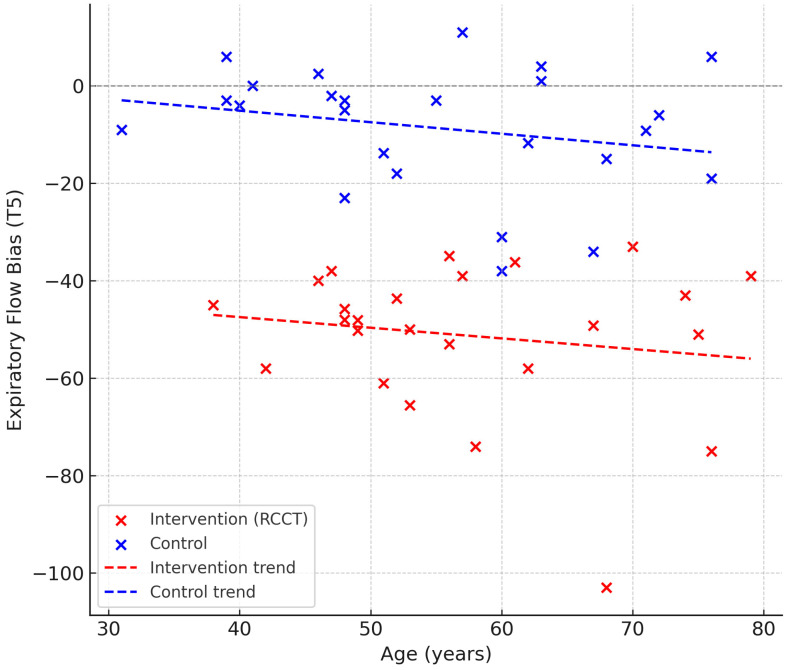
Relationship between age and expiratory flow bias at time point T5 in both study groups.

When analyzing all subjects, males and females displayed significantly different EFB values across all time points (T–5 to T3, *p* < 0.05) (Figure 4). However, within the Intervention group alone, no sex-related differences were observed. Age was not significantly correlated with EFB at any time point, although a weak negative trend was observed in the Control group (ρ = −0.25, *p* = 0.228) (Figure 5).

### 3.4. Lesion Type and Subarachnoid Hemorrhage

Within the Intervention group, no significant differences in EFB were detected across lesion types (Kruskal–Wallis *p* > 0.13 for all comparisons). Similarly, when patients with subarachnoid hemorrhage were compared to other diagnoses, no significant differences in EFB were observed.

### 3.5. Hemodynamic Variables

**Figure 6:**
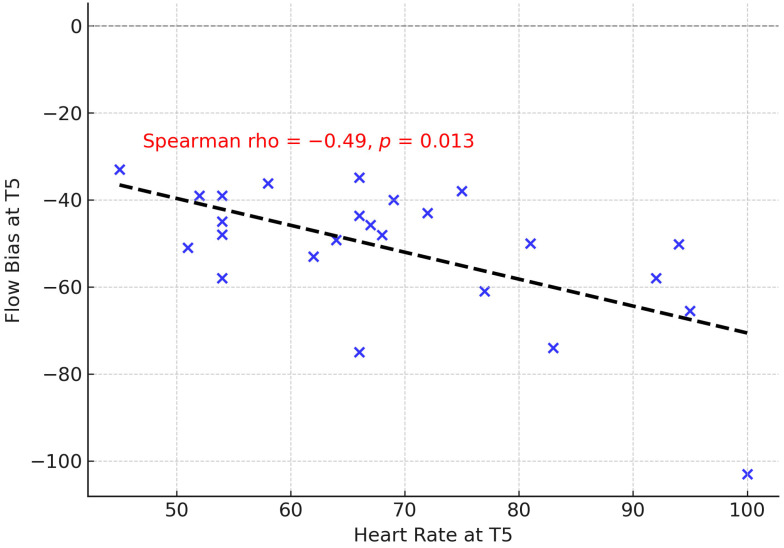
Correlation between heart rate and expiratory flow bias at T5 in the intervention group. Blue crosses represent individual patients, and the dashed line indicates the regression line.

In the Intervention group, mean arterial pressure (MAP) was not significantly correlated with EFB at any time point, although a weak negative association was observed at T0 (ρ = −0.35, *p* = 0.086). Heart rate (HR) also showed no robust associations, except for a moderate negative correlation with EFB at T5 (ρ = −0.49, *p* = 0.013) (Figure 6).

### 3.6. Intracranial Pressure

No significant correlations were found between intracranial pressure (ICP) and EFB at any time point in either group (all *p* > 0.4). Scatterplot analyses confirmed the absence of clear linear or monotonic associations.

### 3.7. Multivariable Analysis

In the multivariable linear regression model (Table 2), allocation to the Intervention group remained the strongest independent predictor of expiratory flow bias at T5 (β = +40.1 L/min, 95% CI 32.5 to 47.7; *p* < 0.001). Higher heart rate was independently associated with lower expiratory flow bias (β = −0.39 per bpm, 95% CI −0.69 to −0.10; *p* = 0.010), and male sex was also negatively associated with flow bias (β = −9.9 L/min, 95% CI −19.2 to −0.5; *p* = 0.039). Age, mean arterial pressure, and lesion type were not significant predictors in the adjusted model. Overall, the model explained 75% of the variability in expiratory flow bias (adjusted R^2^ = 0.75).

### 3.8. Survival Analysis

Patients were recruited between May 2021 and December 2023, and survival status was assessed in September 2024. This provided a follow-up ranging from 9 to 40 months, with a median of approximately 18 months. At this follow-up, survival did not differ significantly between groups. In the intervention group (RCCT), 20 out of 25 patients (80%) were alive compared with 17 out of 25 (68%) in the control group (passive leg mobilization). Although survival was numerically higher in the intervention group, the difference was not statistically significant (χ^2^ = 0.42, *p* = 0.52; Fisher’s exact test *p* = 0.52).

### 3.9. Ventilatory Standardization

At baseline, 12 patients (24%) were on a ventilatory mode different from volume-controlled ventilation and were therefore switched to VC with a square flow pattern one hour before the intervention, as per protocol. This occurred in 7 patients (28%) in the intervention group and 5 patients (20%) in the control group, with no significant difference between groups (Fisher’s exact test, *p* = 0.74).

### 3.10. Exploratory Analyses with Additional Variables

**Figure 7:**
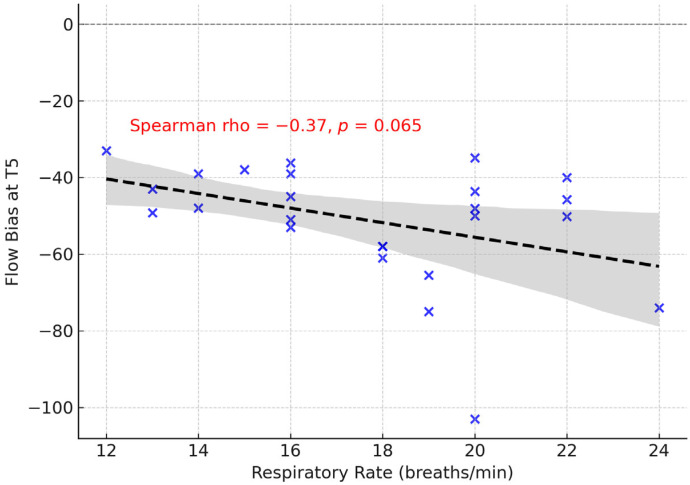
Correlation between respiratory rate and expiratory flow bias at T5 in the intervention group. Blue crosses represent individual patients, the dashed line indicates the regression line, and the shaded area corresponds to the 95% confidence interval.

In the Intervention group, exploratory analyses were performed including smoking status and ventilatory parameters (FiO_2_, PEEP, respiratory rate, and tidal volume). No significant differences in expiratory flow bias (EFB) at T5 were observed between smokers and non-smokers (*p* = 0.92). Similarly, no associations were found between EFB and FiO_2_, PEEP, or tidal volume (all *p* > 0.2). However, respiratory rate showed a moderate negative correlation with EFB (Spearman ρ = −0.37, *p* = 0.065), suggesting a potential trend whereby higher respiratory rates might be associated with reduced expiratory flow bias. Although this did not reach statistical significance, it may indicate a physiologically plausible interaction worthy of further investigation in larger cohorts (Figure 7). Due to missing data, comorbidities could not be included in the exploratory analysis.

## 4. Discussion

This secondary analysis evaluated expiratory flow bias (EFB) in mechanically ventilated neurocritical patients, examining its relationship with patient characteristics, hemodynamic parameters, intracranial pressure (ICP), and arterial blood gases.

Our findings align with previous experimental and clinical studies that identified expiratory flow bias thresholds associated with effective mucus clearance. RCCT consistently generated flow values within or above these ranges, reinforcing its potential as a secretion-mobilizing maneuver. Notably, this physiological effect was achieved in neurocritical patients without adverse changes in intracranial pressure, supporting the safety of its application in this vulnerable population [4,9,16,17].

The immediate difference in expiratory flow bias observed at T0 is explained by the fact that this time point coincided with the initiation of the rapid chest compression technique. The maneuver produces an abrupt expiratory acceleration as soon as it is applied, which accounts for the rapid divergence between groups despite comparable baseline values.

In our cohort, the intervention group demonstrated a significant within-group reduction in PaCO_2_, whereas no change occurred in controls. However, the between-group difference in ΔPaCO_2_ was not significant, and EFB at T5 was not correlated with PaCO_2_ changes. Previous studies of airway clearance techniques have reported similar findings: while interventions such as manual chest compression, ventilator hyperinflation, or PEEP–ZEEP may transiently reduce PaCO_2_ through enhanced alveolar ventilation and secretion mobilization, these effects are often modest and inconsistent across patient populations [17,18,19,20]. In mechanically ventilated patients, short-term PaCO_2_ reductions are typically <5 mmHg and rarely translate into sustained improvements in gas exchange [3,7]. Moreover, in neurocritical care settings, PaCO_2_ is tightly regulated given its strong influence on cerebral blood flow, and any reduction induced by physiotherapy tends to be small and clinically tolerated [7]. Taken together, our results are in line with the literature, suggesting that although RCCT is able to generate substantial expiratory flow bias, its direct impact on PaCO_2_ clearance is limited and should not be considered a primary therapeutic effect of the maneuver.

Sex-related differences in EFB were observed when all patients were analyzed together, but these differences were not present when only the intervention group was considered. This finding likely reflects baseline variability rather than a true treatment interaction. Previous studies have shown that lung size, airway caliber, and expiratory flow rates are generally higher in males than in females when normalized to body size, which may influence flow bias measurements in mechanically ventilated patients [21]. However, clinical trials of chest physiotherapy and airway clearance techniques have not consistently demonstrated sex-specific differences in efficacy [22].

Age was not associated with EFB in our cohort, although a weak, non-significant negative trend was observed in the control group. Age-related changes in respiratory mechanics, such as reduced elastic recoil, increased airway closure, and diminished cough effectiveness, are well documented, and could theoretically impact expiratory flow generation [23]. Nevertheless, our data suggest that within the relatively homogeneous population of severely brain-injured patients, age alone does not substantially influence EFB.

Likewise, lesion type, including subarachnoid hemorrhage, did not appear to modify EFB responses.

Hemodynamic variables showed minimal influence on EFB in our analysis. MAP was not significantly associated at any time point, although a weak negative trend was observed at dif0. A moderate negative correlation between heart rate and EFB at T5 in the intervention group (ρ = −0.49, *p* = 0.013) suggests that tachycardia may impair expiratory flow generation, possibly through altered intrathoracic pressure dynamics. Increases in heart rate are known to reduce diastolic filling time and venous return, which may interact with thoracic pressure swings produced during manual techniques [24]. Previous studies of chest physiotherapy in mechanically ventilated patients have shown transient hemodynamic changes, including rises in heart rate and fluctuations in MAP, but these have generally been modest and clinically well tolerated [25]. Our findings extend this evidence by suggesting a potential inverse relationship between tachycardia and expiratory flow generation, a novel observation that warrants further investigation. Clinically, this may be relevant when applying RCCT in patients with unstable cardiovascular profiles, where excessive heart rate could limit the effectiveness of expiratory bias and secretion clearance.

Importantly, no significant relationship was found between ICP and EFB. This finding reinforces the concept that expiratory flow generation is primarily determined by airway mechanics and ventilatory dynamics rather than by intracranial factors. Previous studies of chest physiotherapy in patients with acute brain injury have reported conflicting effects on ICP, with some describing transient increases during suctioning or percussion [15,19], while others found no clinically relevant changes when manual techniques were applied in isolation [14]. Our results are in agreement with the latter, demonstrating that RCCT can be performed without provoking ICP elevations, consistent with the primary findings of the original trial [14]. The maintenance of ICP stability is particularly relevant in neurocritical care, where secondary brain injury is strongly linked to rises in intracranial pressure. Thus, our data provide further evidence supporting the safety of RCCT as an airway clearance strategy in this vulnerable population.

In our multivariable analysis, allocation to the intervention group was confirmed as the main determinant of increased expiratory flow bias, reinforcing the robustness of the primary finding. Interestingly, two additional independent associations were identified: higher heart rate and male sex were linked to lower expiratory flow bias. The inverse relationship with heart rate may reflect the impact of tachycardia on intrathoracic pressure dynamics and venous return, potentially interfering with expiratory flow generation during physiotherapy maneuvers. Similar hemodynamic–respiratory interactions have been described in studies examining the effects of mechanical ventilation on cardiovascular performance [24]. The negative association observed in males is consistent with physiological reports describing sex-related differences in airway caliber and lung mechanics [21,23]; however, clinical studies of airway clearance techniques have not consistently demonstrated sex-based differences in efficacy [7,22]. These exploratory findings should be interpreted with caution but highlight potential modifiers of expiratory flow bias that warrant further study in larger cohorts.

Taken together, these results reinforce that RCCT can significantly enhance expiratory flow bias in mechanically ventilated neurocritical patients without adversely affecting ICP. This makes the technique not only physiologically relevant but also clinically safe in a population where airway clearance options are limited by neurological vulnerability. As an exploratory secondary analysis, our findings should be interpreted with caution, but they provide valuable new insights and represent, to our knowledge, the first systematic evaluation of RCCT in this patient group. Importantly, they open the way for larger, multicenter studies to assess whether the observed physiological benefits translate into improved clinical outcomes such as reduced pneumonia, shorter ventilation time, and better prognosis [11].

Although a survival analysis was performed, no significant differences were found between groups. These findings should be interpreted with caution, and further studies are needed to explore clinical outcomes.

This analysis has several limitations. The modest sample size limited statistical power, especially for subgroup analyses. The heterogeneity of the neurocritical population and the short-term observation window reduce generalizability. Finally, while EFB is a plausible surrogate of secretion clearance efficacy, this study did not assess clinical outcomes such as pneumonia, duration of mechanical ventilation, or extubation success.

This study assessed the effects of a single RCCT session, and therefore the potential cumulative impact of repeated applications could not be evaluated. Since airway clearance techniques are usually applied multiple times per day in routine practice, future studies should explore whether repeated RCCT sessions maintain effectiveness and influence longer-term outcomes. In addition, we did not perform advanced hemodynamic monitoring such as cardiac ultrasound to evaluate preload or filling pressures. Incorporating these measures in future trials may help to clarify the cardiovascular interactions observed, particularly the inverse relationship between heart rate and expiratory flow bias.

Exploratory analyses incorporating smoking status and ventilatory parameters (FiO_2_, PEEP, respiratory rate, tidal volume) did not identify significant associations with expiratory flow bias, with the exception of a moderate negative trend between respiratory rate and EFB. Although this correlation did not reach statistical significance, it is physiologically plausible, as higher respiratory rates shorten expiratory time and may reduce the capacity to generate expiratory flow bias. This observation should be interpreted cautiously given the limited sample size, but it highlights a potentially relevant interaction that warrants confirmation in larger studies.

## 5. Conclusions

In this secondary analysis of a randomized controlled trial, the rapid chest compression technique significantly increased expiratory flow bias in mechanically ventilated neurocritical patients without provoking adverse intracranial pressure responses. The intervention produced modest reductions in PaCO_2_ but showed no consistent associations with age, sex, lesion type, or mean arterial pressure, while a novel inverse relationship with heart rate was identified. These findings support the physiological safety of RCCT and highlight its potential as an airway clearance strategy in patients where options are limited by neurological vulnerability. Further multicenter studies are warranted to determine whether these physiological benefits may translate into improved patient-centered outcomes.

## Figures and Tables

**Table 1:** Sample description (ABI: acute brain injury) n = 50.

	All Patients		Control Group		Intervention Group		*p* Values
**Age**	**56**		**55**		**57**		0.519
**Time since ABI (mean days)**	**3.3**		**3.3**		**3.3**		**-**
**Sex (% females)**	**33**	66%	**14**	56%	**19**	76%	0.232
**Traumatic brain injury (TBI)**	**12**	24%	**7**	28%	**5**	20%	0.434
**Acute ischemic stroke (AIS)**	**2**	4%	**1**	4%	**1**	4%	0.992
**Subarachnoid hemorrhage (SAH)**	**20**	40%	**8**	32%	**12**	48%	0.798
**Intracerebral hemorrhage (ICH)**	**16**	32%	**9**	36%	**7**	28%	0.434
**Ventricular drainage**	**22**	44%	**9**	36%	**13**	52%	0.396
**Norepinephrine**	**32**	64%	**17**	68%	**15**	60%	1.000
**Decompressive surgery**	**19**	38%	**9**	36%	**10**	40%	0.769
**Relaxation**	**14**	28%	**8**	32%	**6**	24%	0.754

**Table 2:** Multivariable linear regression for predictors of expiratory flow bias at T5.

Variable	β Coefficient (95% CI)	*p*-Value
Group (Intervention vs. Control)	+40.1 (32.5 to 47.7)	<0.001
Age (per year)	−0.27 (−0.60 to +0.07)	0.113
Heart rate (per bpm)	−0.39 (−0.69 to −0.10)	0.010
Mean arterial pressure (per mmHg)	−0.01 (−0.30 to +0.29)	0.971
Sex (Male vs. Female)	−9.9 (−19.2 to −0.5)	0.039
Lesion type: Ischemic stroke	+0.07 (−14.9 to +15.0)	0.992
Lesion type: TBI	−4.1 (−14.7 to +6.4)	0.434

## Data Availability

The datasets generated and analyzed during the current study contain sensitive patient information and are therefore not publicly available due to ethical and legal restrictions. However, the minimal dataset necessary to support the conclusions of this article is available from the corresponding author upon reasonable request. If required by the Editorial Office, the complete raw dataset can be provided in Excel format for confidential internal evaluation.

## Trial Registration

NCT03609866. Registered on 08/01/2018.

## List of Abbreviations

ABI	Acute Brain Injury
ACTs	Airway Clearance Techniques
AIS	Acute Ischemic Stroke
CPP	Cerebral Perfusion Pressure
EFB	Expiratory Flow Bias
ERCC	Expiratory Rib Cage Compression
FiO_2_	Fraction of Inspired Oxygen
GCS	Glasgow Coma Scale
HR	Heart Rate
ICP	Intracranial Pressure
ICH	Intracerebral Hemorrhage
ICU	Intensive Care Unit
MAP	Mean Arterial Pressure
MCC	Manual Chest Compression
MI-E	Mechanical Insufflation–Exsufflation
MV	Mechanical Ventilation
PaCO_2_	Partial Pressure of Carbon Dioxide in Arterial Blood
PaO_2_	Partial Pressure of Oxygen in Arterial Blood
PEEP	Positive End-Expiratory Pressure
PEF	Peak Expiratory Flow
PIF	Peak Inspiratory Flow
RCCT	Rapid Chest Compression Technique
RASS	Richmond Agitation and Sedation Scale
RP	Respiratory Physiotherapy
SAH	Subarachnoid Hemorrhage
SD	Standard Deviation
TBI	Traumatic Brain Injury
VAP	Ventilator-Associated Pneumonia
